# Unveiling Disulfidptosis and Cuproptosis Vulnerabilities in Pterygium: A Transcriptomic Analysis and In-Silico Drug Repurposing Strategy

**DOI:** 10.3390/genes17091105

**Published:** 2026-09-12

**Authors:** İdris Sarıkaya, Recep Kılıç

**Affiliations:** 1 Department of Ophthalmology, Servergazi Hospital, Bereketler, Bereket Cd. No:1, Merkezefendi 20000, Turkey; 2 Department of Family Medicine, Fatih Family Health Center, Fatih, 1905 Sk. No:25, Pamukkale 20170, Turkey; kilicrecep20@gmail.com

**Keywords:** pterygium, disulfidptosis, cuproptosis, transcriptomic analysis, drug repurposing, RNA sequencing

## Abstract

Objectives: Pterygium is an invasive, hyperproliferative fibrovascular disorder of the ocular surface characterized by frequent postoperative recurrence and resistance to apoptosis. This study investigated transcriptional alterations in genes related to the regulated cell death pathways disulfidptosis and cuproptosis in pterygium and explored potential pharmacological candidates through in-silico drug repurposing. Methods: High-throughput RNA-sequencing data (GEO accession GSE155776) from primary pterygium tissues (*n* = 8) and healthy conjunctival controls (*n* = 8) were analyzed. The expression profiles of 15 disulfidptosis-related and 13 cuproptosis-related genes were evaluated. Differential expression signatures were subsequently analyzed using the L1000CDS2 platform in reverse mode to identify perturbagens with transcriptional effects opposing the pterygium-associated signature. Results: Transcriptomic analysis demonstrated marked alterations in actin-cytoskeletal genes, with profound downregulation of *ACTB* and *WASF2* and upregulation of *DSTN* and *CAPZA1*, suggesting a potential vulnerability to disulfide stress. Cuproptosis-related analysis showed increased expression of mitochondrial metabolic genes, including *PDHB* and *DLD*, together with significant overexpression of the copper transporter *SLC31A1*, consistent with transcriptional remodeling of mitochondrial metabolic and copper-handling programs. In-silico drug repurposing identified several FDA-approved drugs with anti-correlative perturbational profiles, including Auranofin, Ouabain, Digitoxin, and Neratinib. Conclusions: Pterygium is associated with coordinated transcriptional alterations involving disulfidptosis- and cuproptosis-related gene networks, suggesting potential structural and metabolic vulnerabilities. In-silico drug repurposing identified several pharmacological candidates whose transcriptional effects oppose the observed disease-associated signature. These findings provide a rationale for further experimental investigation of these pathways and candidate compounds in pterygium.

## 1. Introduction

Pterygium is a prevalent, hyperproliferative, and invasive fibrovascular disorder of the ocular surface, clinically characterized by the wing-shaped encroachment of altered conjunctival tissue onto the cornea [1]. Despite the widespread use of surgical excision techniques, unacceptably high recurrence rates remain a critical clinical challenge, underscoring the urgent need for a comprehensive understanding of its molecular pathogenesis. Pterygium exhibits distinct tumor-like characteristics, including excessive extracellular matrix (ECM) remodeling, epithelial–mesenchymal transition (EMT), and a remarkable evasion of classic programmed cell death mechanisms, particularly apoptosis [2]. This apoptotic resistance allows pterygium fibroblasts and epithelial cells to proliferate uncontrollably, creating a therapeutic imperative to identify and target alternative cell death mechanisms.

Recent breakthroughs in oncology and molecular biology have elucidated novel, non-apoptotic pathways of regulated cell death that hold immense therapeutic potential for hyperproliferative diseases. Disulfidptosis, a newly identified modality of cell death, is triggered by the aberrant accumulation of intracellular disulfide bonds under metabolic stress, which culminates in the catastrophic collapse of the actin cytoskeleton [3]. Given that pterygium pathogenesis is heavily driven by dynamic actin cytoskeleton remodeling, mechanical stress, and myofibroblast differentiation, disulfidptosis represents a highly plausible yet completely unexplored molecular vulnerability within pterygial tissues.

Concurrently, metabolic reprogramming and mitochondrial dysfunction have emerged as critical drivers of fibroproliferative disorders. Cuproptosis, an alternative copper-dependent form of regulated cell death, is characterized by the intracellular accumulation of copper binding directly to lipoylated components of the tricarboxylic acid (TCA) cycle [4]. This interaction induces severe mitochondrial proteotoxic stress. As pterygium tissues are known to adapt to high oxidative demands and metabolic shifts, targeting the cuproptosis pathway offers another promising, yet uninvestigated, therapeutic frontier.

The advent of high-throughput transcriptomics (RNA-seq) paired with advanced computational bioinformatics has revolutionized the discovery of disease-specific genetic signatures. By leveraging established transcriptomic datasets and matching differential expression profiles against massive perturbational databases, such as the Library of Integrated Network-Based Cellular Signatures (LINCS) via the L1000CDS2 search engine, researchers can perform highly accurate in-silico drug repurposing [5]. This data-driven strategy efficiently identifies FDA-approved compounds and bioactive agents capable of reversing pathological gene signatures, significantly bypassing the temporal and financial bottlenecks of traditional de novo drug discovery.

To date, no study has mapped the transcriptomic footprint of disulfidptosis and cuproptosis in pterygium pathogenesis. Therefore, the present study aims to elucidate the expression patterns of these novel cell death pathways using high-resolution RNA-seq data and to identify potential therapeutic candidates via an in-silico drug repurposing approach capable of medically halting pterygium progression and preventing post-surgical recurrence.

## 2. Materials and Methods

### 2.1. Transcriptomic Data Acquisition and Study Design

To investigate transcriptional alterations in genes associated with disulfidptosis and cuproptosis, high-throughput RNA-sequencing data were retrieved from the National Center for Biotechnology Information (NCBI) Gene Expression Omnibus (GEO) database. The dataset (GSE155776) comprises 16 tissue samples: eight primary pterygium specimens and eight healthy conjunctival control specimens. The raw read counts and pre-processed expression matrices were acquired directly from the repository.

According to the original GSE155776 study by Wolf et al. [2], the RNA-sequencing cohort consisted of eight pterygium specimens obtained from eight patients undergoing pterygium surgery and eight healthy conjunctival specimens obtained from eight separate patients undergoing retinal detachment surgery with no history of ocular surface disease. The mean age at surgery was 57.6 ± 8.5 years in the pterygium group and 55.8 ± 7.9 years in the control group, and the male/female distribution was 6/2 in both groups. The specimens were formalin-fixed and paraffin-embedded (FFPE) and analyzed using Massive Analysis of cDNA Ends (MACE), a 3′ RNA-sequencing method. The source study also performed xCell-based cell-type enrichment analysis and demonstrated substantial differences in the cellular microenvironment between pterygium and healthy conjunctiva, including enrichment of myofibroblast-related, stromal, T-lymphocyte, dendritic-cell, and macrophage signatures in pterygium tissue [2].

### 2.2. Identification of Disulfidptosis and Cuproptosis Gene Sets

The core genetic mediators responsible for the execution and regulation of disulfidptosis and cuproptosis were curated based on the most recent foundational literature.

- Disulfidptosis Signature: A panel of 15 key genes regulating actin cytoskeleton dynamics and cellular responses to disulfide stress was analyzed, including *SLC7A11*, *NCKAP1*, *RAC1*, *WASF2*, *CYFIP1*, *ABI1*, *BRK1*, *CAPZA1*, *CAPZB*, *INF2*, *CD2AP*, *ACTB*, *FLNB*, *MYH9*, and *DSTN* [3].
- Cuproptosis Signature: A panel of 13 essential genes involved in mitochondrial lipoylation, the TCA cycle, and copper transport was evaluated, comprising *FDX1*, *LIAS*, *LIPT1*, *DLD*, *DLAT*, *PDHA1*, *PDHB*, *MTF1*, *GLS*, *CDKN2A*, *SLC31A1*, *ATP7B*, and *SLC31A2* [4].

### 2.3. Differential Expression and Statistical Analysis

The differential gene expression analysis between the pterygium and healthy conjunctiva groups was evaluated utilizing the embedded statistical metrics within the GSE155776 dataset. The magnitude of gene expression alterations was quantified using the Base-2 Logarithm of the Fold Change (Log_2_FC). To control the false discovery rate (FDR) in multiple testing, Benjamini–Hochberg-adjusted *p*-values (*p_adj_*) were employed. A gene was considered statistically significant and designated as a Differentially Expressed Gene (DEG) if it met the rigorous threshold of *p_adj_* < 0.05. *ACTB* was included solely as a member of the predefined disulfidptosis-related gene panel and was not used as a housekeeping/reference gene or normalization denominator. It was evaluated using the same differential-expression framework as the other investigated genes.

In the source GSE155776 pipeline, genes with zero reads across all samples were removed before analysis, and gene-level counts were analyzed using DESeq2 (version 1.22.2) with default size-factor normalization, negative-binomial modeling, and Benjamini–Hochberg multiple-testing correction [2]. The source study defined global differentially expressed genes using |Log_2_FC| > 2 and *p_adj_* < 0.05. The present study did not independently reprocess the FASTQ files or rerun differential expression from raw counts; instead, it used the gene-level Log_2_FC and adjusted *p*-values reported in the source DESeq2 (version 1.22.2) output for the targeted gene panels.

For high-resolution data visualization, the transcriptomic distribution of the target cell death-related genes within the global expression profile was mapped using a volcano plot. The horizontal dashed line represents *p_adj_* = 0.05 [−log10(0.05) = 1.301], whereas the vertical dashed lines at Log_2_FC = −1 and +1 are visual effect-size reference lines and were not used as an additional statistical-significance criterion. Concurrently, the directionality and intensity of the significant gene alterations were illustrated via a targeted horizontal Bar Plot. All graphical visualizations and data filtering processes were executed using Python (version 3.10) and the matplotlib (version 3.10.8)/seaborn (version 0.13.2) data visualization libraries.

As an additional gene-set-level analysis, we evaluated whether the multivariate expression profiles of the disulfidptosis- and cuproptosis-related gene panels differed between pterygium and control samples. Sample-level expression values from the processed GSE155776 dataset were log_2_(x + 1)-transformed and standardized gene-wise across samples. Euclidean-distance-based permutational multivariate analysis of variance (PERMANOVA) was performed separately for the 15-gene disulfidptosis-related panel and the 13-gene cuproptosis-related panel. Given the balanced study design (8 pterygium and 8 control samples), statistical significance was evaluated using all 12,870 possible group-label permutations. Homogeneity of multivariate dispersion was additionally assessed using PERMDISP to determine whether significant PERMANOVA results could be attributable to differences in within-group dispersion.

### 2.4. In-Silico Drug Repurposing via L1000cds2

To prioritize pharmacological perturbagens for further experimental evaluation, a signature-based in-silico drug-repurposing strategy was implemented. The most significantly altered genes from the two gene panels were stratified into two directional sets:

- UP-regulated Signature: *DLD*, *PDHB*, *CAPZA1*, *DLAT*, *PDHA1*, *GLS*, *DSTN*, *SLC31A1*, *ABI1.*
- DOWN-regulated Signature: *ACTB*, *WASF2*, *RAC1*, *INF2.*

These directional gene sets were submitted to the L1000CDS2 web-based search engine (Ma’ayan Lab, Icahn School of Medicine at Mount Sinai, New York, NY, USA; maayanlab.cloud/L1000CDS2/), which queries perturbational profiles generated by the NIH LINCS program [5]. The search was performed in “reverse” mode to identify small molecules whose transcriptional effects negatively correlate with the input pterygium-associated signature. L1000CDS2 ranking was interpreted as a measure of transcriptional anti-correlation rather than disease-specific therapeutic efficacy. The returned perturbagens were not interpreted as evidence that they induce disulfidptosis or cuproptosis in pterygium.

## 3. Results

### 3.1. Transcriptomic Landscape of Cell Death Pathways in Pterygium

**Figure 1:**
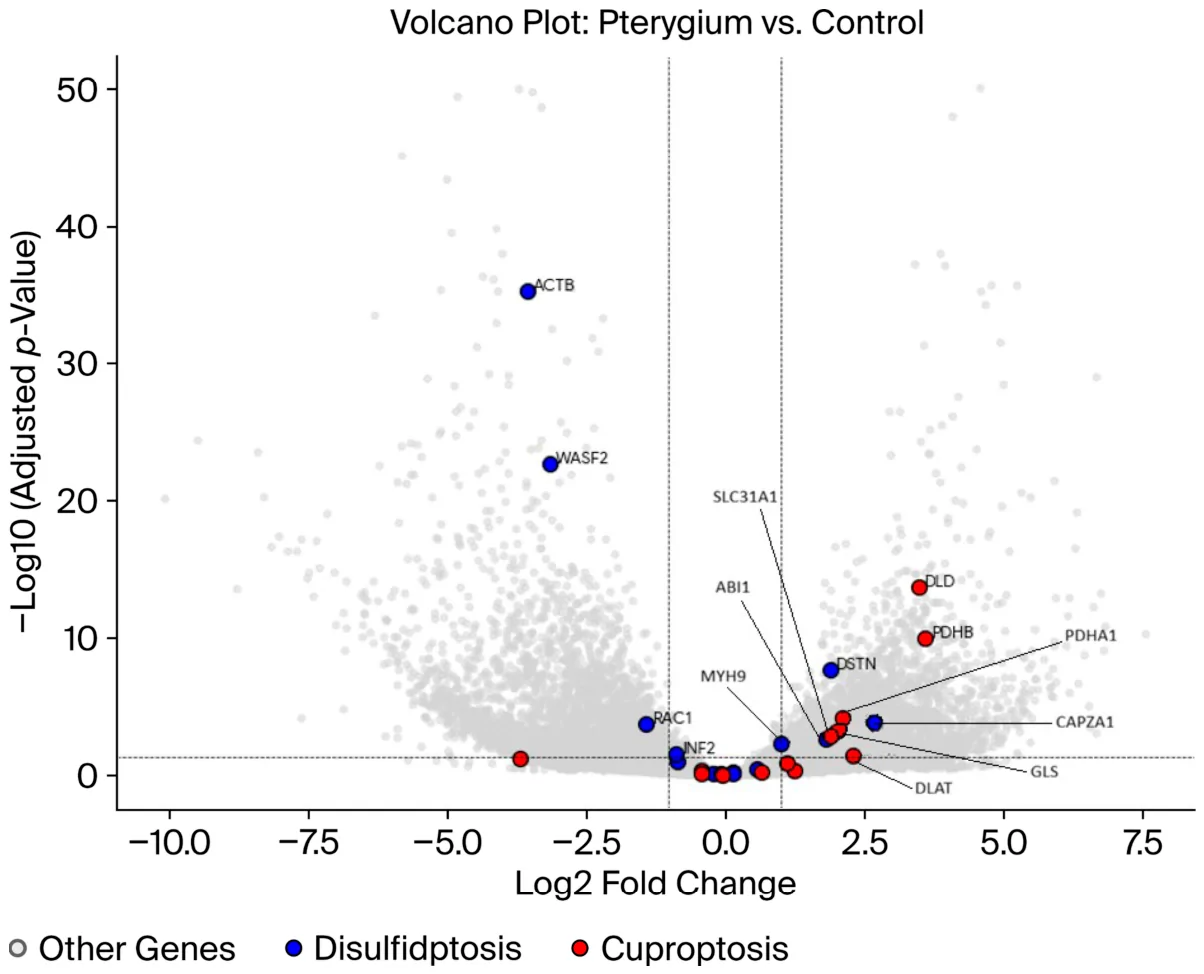
Transcriptomic Distribution of Novel Cell Death Genes in Pterygium vs. Control.

Differential-expression analysis of GSE155776 demonstrated widespread transcriptional differences between pterygium and healthy conjunctival tissues, with numerous genes outside the predefined disulfidptosis- and cuproptosis-related panels showing substantial changes. Within this broader transcriptomic landscape, several genes belonging to the two predefined panels were also significantly dysregulated (Figure 1). These findings identify pathway-associated transcriptional alterations but, by themselves, do not establish a dominant or causal role for disulfidptosis or cuproptosis in pterygium pathogenesis.

We present a volcano plot depicting the global transcriptomic profile of the GSE155776 dataset. The *x*-axis represents Log2 fold change (pterygium/control), and the *y*-axis represents −Log10 adjusted *p*-value. Background genes are shown as semi-transparent grey dots, whereas genes belonging to the predefined disulfidptosis-related and cuproptosis-related panels are highlighted in blue and red, respectively. The horizontal dashed line indicates adjusted *p* = 0.05 [−log10(0.05) = 1.301], and the vertical dashed lines indicate |Log_2_FC| = ±1.

At the gene-set level, the multivariate expression profiles differed significantly between pterygium and control samples for both investigated panels. PERMANOVA demonstrated significant between-group differences for the disulfidptosis-related panel (pseudo-F = 7.88, R^2^ = 0.360, exact permutation *p* = 1.55 × 10^−4^) and the cuproptosis-related panel (pseudo-F = 6.69, R^2^ = 0.323, exact permutation *p* = 1.55 × 10^−4^). PERMDISP showed no significant difference in within-group multivariate dispersion for either the disulfidptosis-related (*p* = 0.610) or cuproptosis-related (*p* = 0.518) panel, indicating that the observed group differences were not attributable to unequal multivariate dispersion. These findings provide gene-set-level evidence of coordinated transcriptional differences involving both panels, without establishing functional activation of either cell-death pathway.

### 3.2. Disulfidptosis-Related Cytoskeletal Transcriptional Alterations

Analysis of the disulfidptosis gene signature demonstrated marked transcriptional alterations in actin-cytoskeletal and mechanotransduction-related genes (Table 1). The most striking transcriptomic suppression was observed in the fundamental cytoskeletal structural components, with *ACTB* (Log_2_FC = −3.55, *p_adj_ =* 5.90 × 10^−36^) and *WASF2* (Log_2_FC = −3.13, *p_adj_ =* 2.18 × 10^−23^) exhibiting massive and statistically significant down-regulation in pterygium tissues. Additionally, *RAC1*, a critical regulator of actin polymerization, was significantly repressed (Log_2_FC = −1.41, *p_adj_ =* 1.85 × 10^−4^).

**Figure 2:**
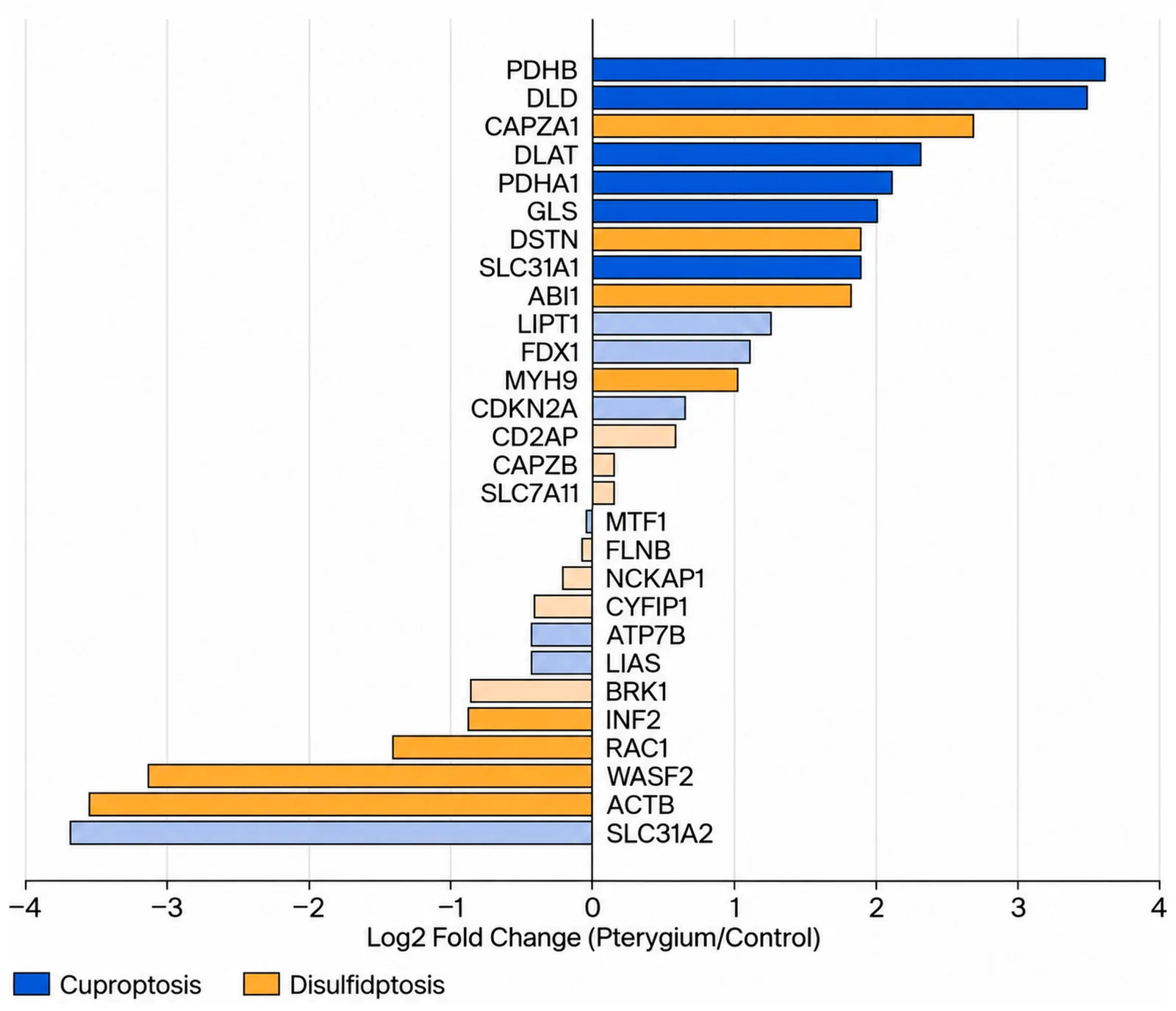
Expression Alterations in Disulfidptosis and Cuproptosis Genes in Pterygium.

Conversely, factors associated with actin depolymerization and cytoskeletal destabilization were strongly up-regulated. Notably, *DSTN* (Log_2_FC = 1.89, *p_adj_ =* 2.29 × 10^−8^) and *CAPZA1* (Log_2_FC = 2.68, *p_adj_ =* 1.45 × 10^−4^) showed highly significant elevations. Other significantly altered genes within this pathway included *ABI1* (Log_2_FC = 1.82, *p_adj_ =* 2.25 × 10^−3^), *MYH9* (Log_2_FC = 1.02, *p_adj_ =* 5.77 × 10^−3^), and *INF2* (Log_2_FC = −0.87, *p_adj_ =* 3.41 × 10^−2^). Notably, *SLC7A11*, an important determinant of disulfidptosis susceptibility in experimental models, was not significantly altered (Log_2_FC = 0.15, *p_adj_* = 0.894). Collectively, these coordinated cytoskeletal alterations suggest a potential vulnerability to disulfide stress and are consistent with transcriptional remodeling of the actin network associated with disulfidptosis; however, they do not by themselves demonstrate functional cytoskeletal collapse or active disulfidptosis in pterygium tissue (Figure 2).

We present a horizontal bar plot illustrating the base-2 logarithm of the fold change (Log_2_FC) for the investigated genes in pterygium tissues compared with healthy conjunctival controls. Orange bars represent disulfidptosis-related genes, whereas blue bars represent cuproptosis-related genes. Faded orange and blue bars indicate transcriptional alterations that did not reach statistical significance (*p_adj_* > 0.05).

### 3.3. Mitochondrial Metabolic Remodeling and Cuproptosis-Related Gene Alterations

Within the cuproptosis-related gene set, several mitochondrial TCA cycle-associated genes were significantly upregulated. The most prominent upregulations among cuproptosis-related mitochondrial metabolic genes were detected in *PDHB* (Log_2_FC = 3.61, *p_adj_* = 1.14 × 10^−10^) and *DLD* (Log_2_FC = 3.48, *p_adj_ =* 2.11 × 10^−14^).

Furthermore, *PDHA1* (Log_2_FC = 2.11, *p_adj_ =* 7.83 × 10^−5^), *GLS* (Log_2_FC = 2.01, *p_adj_ =* 6.81 × 10^−4^), and *DLAT* (Log_2_FC = 2.31, *p_adj_ =* 3.51 × 10^−2^) were also significantly elevated. Notably, the high-affinity copper transporter *SLC31A1* was significantly overexpressed (Log_2_FC = 1.89, *p_adj_* = 1.57 × 10^−3^), suggesting transcriptional alterations in copper homeostasis in pterygium tissue. Together with the upregulation of mitochondrial metabolic genes, this finding is consistent with transcriptional remodeling of copper and mitochondrial metabolic programs, although it does not by itself establish increased copper uptake, cuproptosis activation, or heightened sensitivity to copper-induced cell death (Figure 2).

### 3.4. Candidate Compounds Identified by L1000CDS2

To identify perturbagens with transcriptional effects opposing the selected pterygium-associated signature, the significantly altered genes were submitted to L1000CDS2 in “reverse” mode. The analysis returned FDA-approved drugs, bioactive agents, and tool compounds with anti-correlative perturbational profiles (Table 2).

Among the returned candidates, Auranofin (Rank 9), a thioredoxin reductase inhibitor and redox modulator, was identified as an FDA-approved compound of mechanistic interest. Additionally, cardiac glycosides with known anti-proliferative properties, including Ouabain (Rank 3) and Digitoxin (Rank 4), as well as the potent kinase inhibitor HKI-272/Neratinib (Rank 7), were identified as candidates for experimental prioritization rather than as evidence of therapeutic efficacy in pterygium.

## 4. Discussion

The pathogenesis of pterygium is traditionally characterized by localized limbal stem cell dysfunction, chronic ultraviolet (UV) exposure, and a robust fibrovascular proliferative response. Because pterygial cells exhibit aggressive, tumor-like behaviors—such as EMT, ECM remodeling, and profound resistance to classical apoptosis—pterygium is frequently described as a benign neoplastic lesion [6]. While the evasion of apoptosis by pterygial fibroblasts is well-documented and recognized as a primary cause of high postoperative recurrence rates [7,8], therapeutic strategies targeting alternative, non-apoptotic cell death modalities remain largely unexplored in ophthalmology. To the best of our knowledge, the present study is the first to investigate transcriptional alterations in genes related to two novel forms of regulated cell death—disulfidptosis and cuproptosis—in pterygium. By integrating high-resolution RNA-seq data with the L1000CDS2 perturbation database, we identified pathway-associated transcriptional patterns and prioritized a panel of candidate compounds for further pharmacological investigation.

### 4.1. Cytoskeletal Remodeling, EMT, and Potential Disulfidptosis Vulnerability

Disulfidptosis is a recently defined, actin-dependent cell-death mechanism in which disulfide stress can destabilize the F-actin network under specific metabolic conditions [3]. In the present dataset, several actin-regulatory genes within the disulfidptosis-related panel were altered, most notably *ACTB* (Log_2_FC = −3.55), *WASF2* (Log_2_FC = −3.13), and *RAC1* (Log_2_FC = −1.41), whereas actin depolymerization factors such as *DSTN* and *CAPZA1* were significantly upregulated. This coordinated but directionally heterogeneous pattern is consistent with substantial remodeling of actin-cytoskeletal regulation in pterygium.

The recent literature highlights EMT as an important process in pterygium progression and recurrence [9]. EMT-associated cell migration and invasion involve dynamic remodeling of the actin cytoskeleton. The reorganization of the actin network is not merely a structural necessity but a critical signaling hub for cellular motility. The marked down-regulation of *ACTB* and *RAC1* (Log_2_FC = –1.41) observed in our results is consistent with substantial alteration in cytoskeletal regulation in pterygium tissue; however, these transcriptional changes alone cannot establish impaired mechanical or structural integrity at the functional level. Importantly, *ACTB* was included in the present analysis as a disulfidptosis-related gene and was not used as a housekeeping/reference gene or for normalization. Given the bulk-tissue nature of GSE155776 and the reported differences in cellular composition between pterygium and healthy conjunctiva [2], the marked *ACTB* alteration may reflect transcriptional regulation, differences in cellular composition, or a combination of both. Furthermore, studies on aggressive fibrotic diseases have demonstrated that hyperproliferative cells with high actin turnover may exhibit increased vulnerability to disulfide stress when glucose metabolism is perturbed [3]. In this context, the expression pattern observed in pterygium raises the possibility of a disulfidptosis-related cytoskeletal vulnerability but does not demonstrate that pterygium tissue is intrinsically susceptible to disulfidptosis. Moreover, *SLC7A11*, an important determinant of disulfidptosis susceptibility in experimental models, was not significantly altered in this dataset, indicating that the observed transcriptional pattern represents only partial overlap with the canonical disulfidptosis network. Pharmacologically targeting this cytoskeletal stress response may therefore warrant further investigation as a potential strategy to modulate the migratory behavior of pterygium fibroblasts, although functional validation is required before therapeutic implications can be established [10,11].

### 4.2. Mitochondrial Metabolic Alterations and Cuproptosis

Cuproptosis, distinct from oxidative stress-induced ferroptosis or apoptosis, is driven by copper binding directly to lipoylated enzymes within the mitochondrial TCA cycle, resulting in irreversible proteotoxic stress and cell death [4]. In the present analysis, several genes within the cuproptosis-related panel were upregulated, including *DLD* (Log_2_FC = 3.48), *PDHB* (Log_2_FC = 3.61), *PDHA1, DLAT, GLS*, and the copper transporter *SLC31A1*. These alterations are consistent with changes in mitochondrial metabolic and copper-handling programs in pterygium but cannot establish cuproptotic flux or copper-dependent cell death.

The observed transcriptomic elevations suggest alterations in mitochondrial metabolic activity in pterygium that may support its proliferative and tumor-like biological features. Similar metabolic reprogramming, including shifts in mitochondrial and TCA cycle metabolism, has been extensively documented in neoplastic and fibroproliferative disorders and has been associated with altered susceptibility to copper-dependent cell death mechanisms [12,13]. Importantly, the significant overexpression of the high-affinity copper transporter *SLC31A1* (Log_2_FC = 1.89) observed in our study suggests altered copper handling in pterygium tissue. Recent oncological data have demonstrated that *SLC31A1* upregulation enhances cellular copper uptake and promotes CPT1A-associated fatty acid oxidation, ATP production, and proliferative activity in cancer cells [14]. In this context, increased *SLC31A1* expression in pterygium may similarly reflect altered copper-dependent metabolic programming that contributes to its hyperproliferative phenotype. Nevertheless, the cuproptosis-related transcriptional pattern was only partially concordant, as several canonical pathway-associated genes, including *FDX1*, *LIAS*, *LIPT1*, *ATP7B*, and *MTF1*, were not significantly altered. Therefore, these findings are more appropriately interpreted as partial transcriptional remodeling involving mitochondrial metabolism and copper handling rather than evidence of active cuproptosis or established susceptibility to copper-induced cell death. Copper ionophores or TCA cycle modulators may nevertheless represent candidates for future experimental investigation of cuproptosis-related vulnerabilities in pterygium, although their ability to selectively induce cuproptosis in pterygium cells while sparing healthy conjunctival tissue requires direct functional validation. The additional gene-set-level multivariate analysis further demonstrated significant overall differences in the expression profiles of both the disulfidptosis- and cuproptosis-related panels between pterygium and control tissues. These findings support coordinated transcriptional differences at the gene-set level but should not be interpreted as evidence of functional activation of either cell-death pathway.

When the two gene sets are considered as coordinated modules rather than as isolated genes, the data suggest concurrent remodeling of cytoskeletal, metabolic, and redox-associated programs. Within the disulfidptosis-related panel, *ACTB*, *WASF2*, and *RAC1* were downregulated, whereas *DSTN*, *CAPZA1*, *ABI1*, and *MYH9* were upregulated. In parallel, several cuproptosis-related genes involved in mitochondrial metabolism and copper handling, including *DLD*, *PDHB*, *PDHA1*, *DLAT*, *GLS*, and *SLC31A1*, were upregulated. Taken together, this coordinated but heterogeneous expression pattern may reflect concurrent cellular adaptations involving cytoskeletal organization, metabolic activity, and redox homeostasis in pterygium. However, bulk RNA-sequencing data cannot establish direct gene–gene regulatory relationships, pathway cross-talk, or the causal sequence of these alterations.

### 4.3. Therapeutic Implications and In-Silico Drug Repurposing

The identification of therapeutic candidates capable of counteracting the pterygium-associated transcriptional signature represents a potentially clinically relevant outcome of this study. Through our L1000CDS2 in-silico perturbational screening, Auranofin emerged as one of the FDA-approved candidate compounds identified by the analysis. Originally developed as an oral anti-rheumatic agent, Auranofin is a potent inhibitor of thioredoxin reductase (TrxR), a key enzyme involved in maintaining intracellular redox homeostasis [15]. In the context of disulfide-stress biology, inhibition of the thioredoxin system can impair cellular reducing capacity and promote oxidative and disulfide stress, processes that may influence actin cytoskeletal integrity under conditions associated with disulfidptosis [16]. Furthermore, Auranofin has shown antifibrotic effects in experimental models of liver fibrosis, where it suppressed fibrogenesis through inhibition of the NLRP3 inflammasome [17], providing a mechanistic rationale for further investigation in the context of redox- and disulfide-stress biology. However, the present analysis does not demonstrate that Auranofin induces disulfidptosis in pterygium cells, nor does it establish an effective or safe topical concentration. These questions require direct dose–response and mechanistic experiments in primary pterygium cells and appropriate in-vivo models. Although Auranofin ranked ninth by overlap score, it was highlighted for mechanistic discussion because of its FDA-approved status and its established TrxR/redox mechanism, which provides direct biological relevance to the disulfide-stress axis. This emphasis reflects biological plausibility rather than a higher L1000CDS2 rank or a separate computational re-ranking procedure.

Furthermore, the identification of cardiac glycosides (Ouabain and Digitoxin) and the kinase inhibitor Neratinib (HKI-272) provides additional candidates for therapeutic investigation. Cardiac glycosides bind to Na+/K+-ATPase and can trigger downstream signaling pathways associated with apoptosis and autophagy in cancer cells [18]. Previous in-vitro studies have also reported anti-fibrotic effects of Ouabain, including inhibition of myofibroblast-associated responses, providing biological support for its further evaluation [19]. Neratinib targets EGFR/HER2 signaling pathways that are relevant to growth factor-mediated proliferation, cell migration, and fibrotic processes [20]. Nevertheless, these compounds were identified through perturbational profiles generated in non-pterygium cell lines, and the present analysis does not establish their therapeutic efficacy or mechanism of action in pterygium. Their relevance should therefore be considered hypothesis-generating and requires direct validation in pterygium-specific experimental models.

### 4.4. Limitations and Future Directions

Several limitations should be considered when interpreting the present findings. First, this study is a secondary, hypothesis-driven analysis of a publicly available transcriptomic dataset rather than an independent experimental cohort. The 28 genes examined here constitute only a small subset of the extensive transcriptional alterations occurring in pterygium. In the original analysis of GSE155776, 1881 genes were upregulated and 1676 were downregulated according to the source study’s DEG criteria [2]. Therefore, differential expression of selected disulfidptosis- and cuproptosis-related genes should not be interpreted as evidence that these pathways are dominant or central drivers of pterygium pathogenesis. Second, GSE155776 is based on bulk RNA sequencing of whole-tissue specimens. The original study demonstrated substantial differences in the cellular microenvironment between pterygium and healthy conjunctiva, including enrichment of myofibroblast-related, stromal, T-cell, dendritic-cell, and macrophage signatures in pterygium tissue [2]. Consequently, differences in genes such as *ACTB* may reflect transcriptional regulation within cells, changes in cellular composition, or a combination of both. Bulk RNA sequencing cannot distinguish these possibilities. In addition, although group-level age and sex distributions were similar, patient-level clinical characteristics were not modeled in the present secondary analysis; residual clinical heterogeneity may therefore contribute to the observed expression differences. Third, differential mRNA expression does not establish functional activation of disulfidptosis or cuproptosis. Several canonical genes associated with these pathways were not significantly altered, and the dataset does not provide direct measurements of disulfide accumulation, F-actin collapse, intracellular copper, protein lipoylation, proteotoxic stress, or pathway-specific cell death. The present findings should therefore be considered hypothesis-generating pathway-associated transcriptional signatures requiring experimental validation. Finally, L1000CDS2 predictions are based on perturbational profiles generated in heterogeneous human cell lines rather than primary pterygium cells. The identified compounds therefore represent candidates for experimental prioritization rather than validated therapies. Future studies should validate these findings in independent cohorts and, where feasible, single-cell or spatial transcriptomic datasets, followed by mechanistic experiments in primary human pterygium cell cultures and appropriate in-vivo models to evaluate pathway activity, pharmacological response, safety, and dose requirements.

## 5. Conclusions

In summary, our study provides initial transcriptomic evidence that pterygium is associated with alterations in disulfidptosis- and cuproptosis-related gene networks. The profound suppression of actin cytoskeletal genes and the increased expression of mitochondrial metabolic genes suggest distinct metabolic and structural vulnerabilities. Through in-silico repurposing, we identified several FDA-approved agents, including Auranofin, as candidate compounds predicted to oppose the observed transcriptional signature. These findings provide a translational rationale for further experimental evaluation of topical pharmacological strategies in pterygium, alongside current surgical approaches.

## Figures and Tables

**Table 1:** Differential Expression Profile of Target Cell Death Genes in Pterygium

Gene Symbol	Pathway	Mean (Ptx)	Mean (Control)	Log_2_FC	*p*-Value	Adjusted *p*-Value
ACTB	Disulfidptosis	66.77	779.26	−3.55	9.37 × 10^−39^	5.90 × 10^−36^
WASF2	Disulfidptosis	25.61	228.40	−3.13	1.24 × 10^−25^	2.18 × 10^−23^
DSTN	Disulfidptosis	112.75	29.82	1.89	1.43 × 10^−9^	2.29 × 10^−8^
CAPZA1	Disulfidptosis	18.23	2.95	2.68	2.39 × 10^−5^	1.45 × 10^−4^
RAC1	Disulfidptosis	34.57	92.77	−1.41	3.14 × 10^−5^	1.85 × 10^−4^
ABI1	Disulfidptosis	19.73	5.87	1.82	5.43 × 10^−4^	2.25 × 10^−3^
MYH9	Disulfidptosis	73.47	36.28	1.02	1.62 × 10^−3^	5.77 × 10^−3^
INF2	Disulfidptosis	19.05	35.64	−0.87	1.31 × 10^−2^	3.41 × 10^−2^
BRK1	Disulfidptosis	10.64	19.74	−0.86	4.85 × 10^−2^	9.89 × 10^−2^
CD2AP	Disulfidptosis	12.85	8.90	0.59	2.99 × 10^−1^	4.11 × 10^−1^
CAPZB	Disulfidptosis	44.94	39.80	0.16	5.75 × 10^−1^	6.79 × 10^−1^
NCKAP1	Disulfidptosis	18.67	21.69	−0.21	6.85 × 10^−1^	7.70 × 10^−1^
FLNB	Disulfidptosis	42.72	44.87	−0.08	8.33 × 10^−1^	8.84 × 10^−1^
SLC7A11	Disulfidptosis	2.65	2.60	0.15	8.47 × 10^−1^	8.94 × 10^−1^
CYFIP1	Disulfidptosis	0.00	0.22	−0.41	8.93 × 10^−1^	N/A
DLD	Cuproptosis	36.34	3.48	3.48	3.88 × 10^−16^	2.11 × 10^−14^
PDHB	Cuproptosis	26.24	2.07	3.61	4.46 × 10^−12^	1.14 × 10^−10^
PDHA1	Cuproptosis	28.26	6.29	2.11	1.20 × 10^−5^	7.83 × 10^−5^
GLS	Cuproptosis	21.71	5.73	2.01	1.39 × 10^−4^	6.81 × 10^−4^
SLC31A1	Cuproptosis	17.90	4.59	1.89	3.60 × 10^−4^	1.57 × 10^−3^
DLAT	Cuproptosis	7.79	1.65	2.31	1.36 × 10^−2^	3.51 × 10^−2^
SLC31A2	Cuproptosis	0.00	3.02	−3.68	2.80 × 10^−2^	6.36 × 10^−2^
FDX1	Cuproptosis	10.29	5.03	1.11	6.81 × 10^−2^	1.30 × 10^−1^
LIPT1	Cuproptosis	3.21	1.42	1.26	3.57 × 10^−1^	4.73 × 10^−1^
LIAS	Cuproptosis	8.70	11.66	−0.42	3.76 × 10^−1^	4.92 × 10^−1^
CDKN2A	Cuproptosis	5.66	3.37	0.65	4.52 × 10^−1^	5.67 × 10^−1^
ATP7B	Cuproptosis	2.20	3.10	−0.42	6.84 × 10^−1^	7.69 × 10^−1^
MTF1	Cuproptosis	5.42	5.50	−0.04	9.43 × 10^−1^	9.61 × 10^−1^

Summary of the statistical expression data for the investigated disulfidptosis- and cuproptosis-related genes. Genes are grouped by gene panel and sorted by their adjusted *p*-values. Ptx indicates the pterygium group, and Log_2_FC represents the base-2 logarithm of fold change. Statistical significance was defined as *p_adj_* < 0.05.

**Table 2:** Selected Perturbagens Identified by L1000CDS2 Reverse-Signature Analysis

Rank	Compound/Perturbation	Target Mechanism/Drug Class	Overlap Score	Tested Cell Line	Dose (μM)
1	BRD-K12184916	Tool Compound	0.2308	NCIH508	0.63
2	NSC 632839	Antineoplastic/Tool Compound	0.1538	HCC515	10.0
3	Ouabain	Cardiac Glycoside/Anti-proliferative	0.1538	HCC515	10.0
4	Digitoxin	Cardiac Glycoside/Anti-proliferative	0.1538	HCC515	10.0
5	Erythrosine Sodium	Food Dye/Bioactive Agent	0.1538	HCC515	10.0
7	HKI-272 (Neratinib)	EGFR/HER2 Kinase Inhibitor	0.1538	A375	10.0
9	Auranofin	TrxR Inhibitor/Redox Modulator	0.1538	A549	10.0
11	PX12	Thioredoxin-1 (Trx-1) Inhibitor	0.1538	COV644	30.0
12	Methylene Blue	Redox Indicator/Dye	0.1538	HCC15	10.0

Selected perturbagens returned by the LINCS L1000/L1000CDS2 reverse-signature analysis. The table includes FDA-approved drugs, bioactive agents, and tool compounds. Rankings and overlap scores indicate anti-correlative perturbational matching to the input pterygium-associated gene signature.

## Data Availability

The datasets analyzed during the current study are publicly available in the National Center for Biotechnology Information (NCBI) Gene Expression Omnibus (GEO) repository under accession number GSE155776. Additional data generated during the current study are available from the corresponding author upon reasonable request.
